# Membrane Vesicles from *Lactobacillus acidophilus* Promote Superior Cytokine Modulation and Antimicrobial Signaling Compared with Their Whole Cells in RAW 264.7 Macrophages

**DOI:** 10.3390/ijms27062764

**Published:** 2026-03-18

**Authors:** Cristal Dafne Lonngi Sosa, Francisco Rodolfo González Díaz, Hugo Ramírez Álvarez, Alejandro Vargas Ruiz, Rosa Isabel Higuera Piedrahita, Héctor Alejandro de la Cruz Cruz, Jorge Alfredo Cuéllar Ordaz, Gerardo Ramírez-Rico, Erasmo Negrete Abascal, Magda Reyes López, Cynthia González Ruíz

**Affiliations:** 1 Unidad de Investigación Multidisciplinaria, Facultad de Estudios Superiores Cuautitlán, Universidad Nacional Autónoma de México (UNAM), Carretera Cuautitlán-Teoloyucan Km 2.5, Col. San Sebastián Xhala, Cuautitlán Izcalli 54714, Mexico; skriztal@gmail.com (C.D.L.S.); folodro2013@gmail.com (F.R.G.D.); ramiralh@unam.mx (H.R.Á.); styfler18@hotmail.com (A.V.R.); rositah_10@cuautitlan.unam.mx (R.I.H.P.); delacruz@unam.mx (H.A.d.l.C.C.); jcuellar@unam.mx (J.A.C.O.); garmvz@gmail.com (G.R.-R.); 2 Facultad de Estudios Superiores Iztacala, Universidad Nacional Autónoma de México (UNAM), Tlalnepantla de Baz 54090, Mexico; negretee@yahoo.com; 3 Departamento de Biología Celular, Centro de Investigación y de Estudios Avanzados del IPN, Ciudad de México 07360, Mexico; magda.magrel.2003@gmail.com

**Keywords:** membrane vesicles, *Lactobacillus acidophilus*, cytokine modulation, whole cells, antimicrobial signaling

## Abstract

The interaction between probiotic bacteria and the innate immune system is of increasing interest due to its capacity to modulate inflammatory and antimicrobial responses. The murine macrophage cell line RAW 264.7 is widely used to investigate the immunomodulatory effects of probiotic bacteria and their cell-free derivatives, such as membrane vesicles (MVs). In this study, we evaluated whether MVs derived from *Lactobacillus acidophilus* promote superior modulation of cytokine production and antimicrobial signaling in RAW 264.7 macrophages compared with whole cells (WCs). Our results show that *L. acidophilus* MVs exhibited direct bactericidal activity against *Escherichia coli* and induced a more selective and balanced cytokine profile than whole cells. These findings highlight the potential of probiotic-derived membrane vesicles as acellular immunomodulatory effectors for the development of novel cell-free biotherapeutic strategies.

## 1. Introduction

The interaction between probiotic bacteria and the innate immune system has garnered considerable interest due to its potential to modulate inflammatory and antimicrobial responses in mucosal epithelial tissues [1,2]. In this context, macrophages play a crucial role in the mucosal immune response by phagocytosing pathogens, maintaining intestinal epithelial homeostasis, and presenting antigens to immune cells. They also orchestrate immune signaling through the recognition of microorganism-associated molecular patterns via pattern recognition receptors (PRRs), including Toll-like receptors (TLRs). This recognition regulates cytokine production, promotes antibody responses, and enhances nonspecific immune defense mechanisms [3,4]. To investigate these mechanisms in vitro, the murine macrophage cell line RAW 264.7 has been widely used as an experimental model to study the immunomodulatory effects induced by probiotics and their cell-free derivatives, including membrane vesicles (MVs) [5]. MVs are spherical structures actively secreted during all phases of bacterial growth by both Gram-positive and Gram-negative bacteria. In general, these bacterial vesicles range from 40 to 400 nm in diameter [6] and transport a diverse array of biologically active molecules—such as membrane proteins, lipids, polysaccharides, toxins, and nucleic acids—that can interact with host cells and mediate specific immune responses [7,8]. In this context, it has been reported that the administration of MV transport and delivery concentrates microbial antigens on the target, thereby avoiding some of the risks associated with the administration of whole bacterial cells (WCs), including excessive inflammatory stimulation or systemic bacterial translocation [9].

*Lactobacillus acidophilus* is one of the most extensively studied probiotic species and has demonstrated beneficial immunomodulatory effects in both animal models and cell-based systems, including the regulation of proinflammatory and anti-inflammatory cytokines and the enhancement of innate antimicrobial mechanisms [10,11]. Experimental evidence indicates that *L. acidophilus* MVs activate RAW 264.7 macrophages, inducing morphological changes associated with cellular activation and significantly increasing the expression of proinflammatory cytokines, such as IL-1β and TNF-α [12,13]. This activation profile indicates that MVs can elicit a robust immune response that may be modulated more effectively and with greater control than stimulation with whole bacteria. This has been demonstrated in studies using probiotic vesicles, such as those derived from *Lactobacillus helveticus*, which modulate cytokine production in RAW 264.7 cells [14], as well as in investigations describing comparable immunomodulatory mechanisms induced by probiotic *Escherichia coli* Nissle 1917-derived outer membrane vesicles [15].

The aim of the present study was to determine whether MVs derived from *Lactobacillus acidophilus* exert superior immunomodulatory and antimicrobial effects against *Escherichia coli* compared with their WCs. To this end, bacterial inhibition assays were performed in a semi-solid medium inoculated with *E. coli*. Following confirmation of their antimicrobial activity, RAW 264.7 macrophages were stimulated with MVs or WCs of *L. acidophilus* and subsequently challenged with *E. coli*. The immune response was assessed by analyzing the expression of cytokines and receptors, including IL-1β, TNF-α, IL-10, IL-12, and TLR2. Our findings demonstrate that MVs obtained from *L. acidophilus* isolated from the ileum of free-living rats exhibited both direct bactericidal activity against *Escherichia coli* and the capacity to modulate macrophage immune responses. In comparison with WCs, MVs induced a more selective and balanced cytokine profile, characterized by the coordinated expression of proinflammatory and regulatory mediators. Collectively, these results suggest that MVs from *L. acidophilus* act as acellular effectors with a distinctive ability to modulate cytokine production and activate antimicrobial signaling pathways in RAW 264.7 macrophages. A deeper understanding of the transport and delivery of antigenic molecules via membrane vesicles highlights their potential as a versatile biological platform. Such acellular systems may overcome some of the limitations associated with conventional probiotic therapies by enabling more targeted immunomodulation while simultaneously opening new perspectives for the development of therapeutic strategies based on microbial-derived vesicles, with applications in immunology, inflammatory diseases, and antimicrobial resistance.

## 2. Results

### 2.1. Lactobacillus acidophilus Isolated from the Ileum of Free-Living Rats Releases MVs

**Figure 1:**
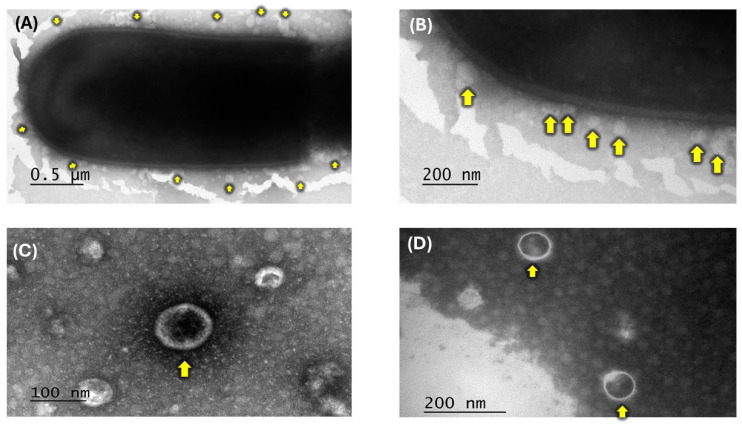
Negative-stained transmission electron microscopy of *L. acidophilus* membrane vesicles (MVs) is shown with yellow arrows. (**A**) The formation of multiple MVs surrounding the peptidoglycan layer of the bacterial cell. (**B**) Close-up of the peptidoglycan layer with spherical MVs. (**C**) MVs show an approximate diameter of 100 nm. (**D**) MVs (yellow arrows) show a bilayer and a diameter less than 200 nm.

To characterize and confirm the release of membrane vesicles (MVs) from *Lactobacillus acidophilus* isolated from the ileum of free-living rats, transmission electron microscopy (TEM) was performed. Negative-staining TEM analysis revealed the formation of multiple spherical MVs surrounding the peptidoglycan layer of *L. acidophilus* (Figure 1A,B). Close-up images further demonstrated the double membrane of the vesicles and a diameter of 100–200 nm (Figure 1C,D).

### 2.2. Antimicrobial Effect of L. acidophilus MVs Is Higher than That of Their Whole Cells (WCs) Against Escherichia coli

**Figure 2:**
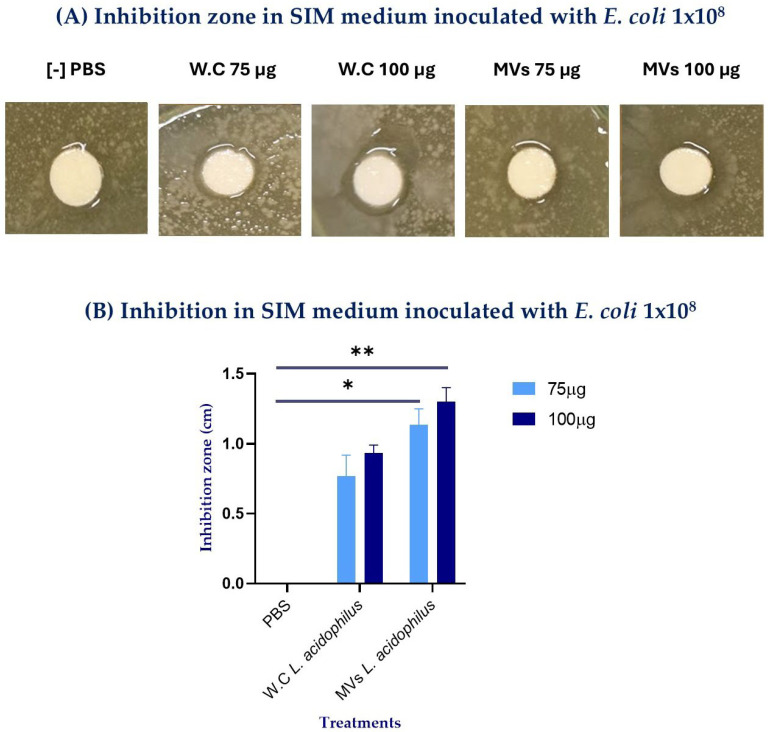
Inhibition assay performed in SIM medium inoculated with *E. coli* (1 × 10^8^) after the addition of different concentrations of whole cells (WCs) or membrane vesicles (MVs) derived from *Lactobacillus acidophilus*. (**A**) Representative inhibition zones observed after treatment with PBS and 75 or 100 μg of WCs or MVs. (**B**) Quantification of inhibition zones obtained with different concentrations of WCs and MVs. Statistical analysis was performed using one-way ANOVA. Significance is indicated as * *p* ≤ 0.1 and ** *p* ≤ 0.05.

After isolation of MVs by ultrafiltration and ultracentrifugation and subsequent morphological verification by transmission electron microscopy (TEM), their antimicrobial activity was assessed using disk diffusion assays. The size of the inhibition halos was dependent on the total protein content of the samples (75 and 100 μg of total protein) for both MVs and WCs, with the most significant effect observed at 100 μg of protein (Figure 2A). Notably, 100 μg of *L. acidophilus* MVs produced significantly greater inhibition compared with PBS (** *p* = 0.043). In contrast, WCs at the same protein concentration showed a minor difference compared to PBS (* *p* = 0.062) (Figure 2B).

**Figure 3:**
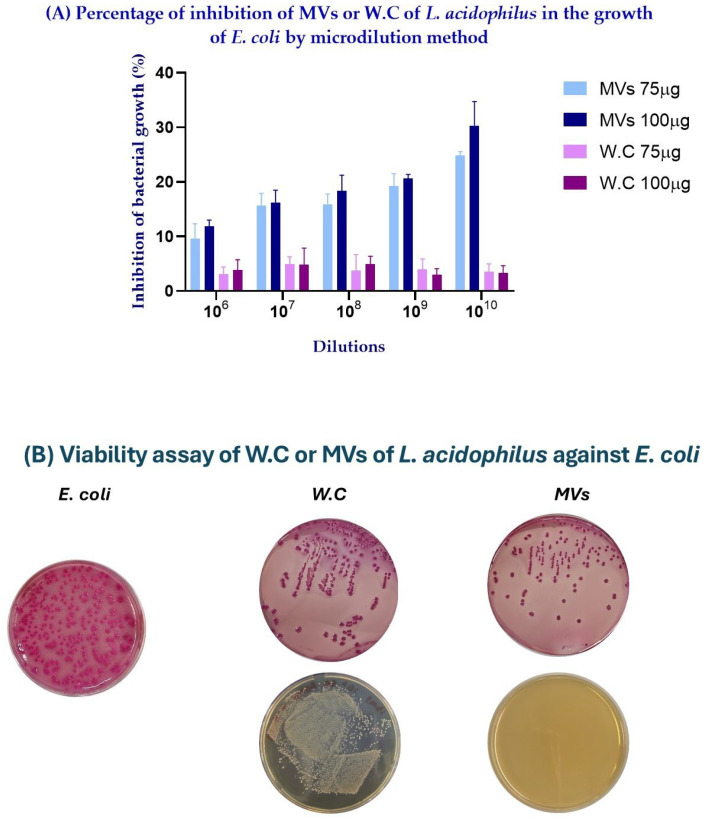
Inhibitory effect of membrane vesicles (MVs) and whole cells (WCs) of *Lactobacillus acidophilus* on *Escherichia coli* growth evaluated by microdilution assay and viability testing. (**A**) Percentage of inhibition produced by 75 or 100 μg of MVs or WCs at different *E. coli* loads. Positive and negative controls are not shown, as they corresponded to 100% and 0% inhibition, respectively. (**B**) Representative images of the viability assays performed on MacConkey agar for *E. coli* and on MRS agar for *L. acidophilus* WCs or MVs at an *E. coli* load of 10^10^.

To further evaluate the antibacterial activity of MVs under different bacterial loads, a microdilution assay was performed. Consistent with the inhibition assays, MVs showed a dose-dependent inhibitory effect against *E. coli*, with the highest inhibition observed at the highest MV concentrations. In contrast, WCs showed a lower inhibitory effect compared with MVs, and the percentage of inhibition decreased as the concentration of WCs increased (Figure 3A).

To further investigate this observation, a bacterial viability assay was performed. Samples were plated on MacConkey agar to evaluate the viability of *E. coli*, whereas MRS agar was used to assess the viability of *L. acidophilus* WCs. On MacConkey agar, a reduction in *E. coli* viability was observed with increasing dilution in both the MVs- and WC-treated samples. However, on MRS agar, higher concentrations of WCs resulted in increased bacterial growth, whereas no growth was observed in samples treated with MVs (Figure 3B). These results suggest that the absorbance values and calculated percentage of inhibition in the WC treatments were influenced by the simultaneous growth of *L. acidophilus* WCs and *E. coli* in the microdilution assay.

### 2.3. Administration of WCs and MVs of L. acidophilus Triggers Activation of RAW 264.7 Cells

**Figure 4:**
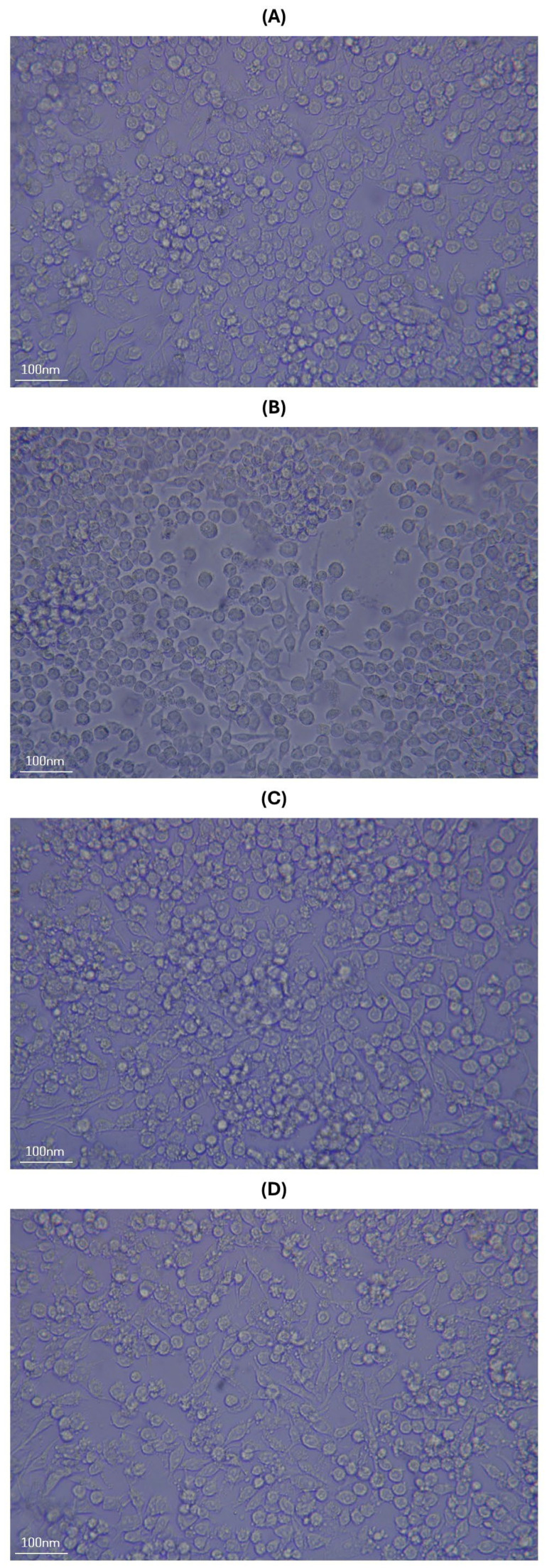
Morphological changes observed in RAW 264.7 cells using an inverted light microscope with a 40× objective (total magnification, 400×) at 5 h after stimulation at 0, 2, and 4 h with the different treatments: (**A**) PBS-stimulated cells; (**B**) cells stimulated with 2 μg of commercial *Escherichia coli* LPS; (**C**) cells stimulated with 10 μg of whole cells (WCs) of *L. acidophilus*; and (**D**) cells stimulated with 10 μg of membrane vesicles (MVs) of *L. acidophilus*.

Once the antimicrobial activity of *Lactobacillus plantarum* MVs was confirmed, their impact on the immune response was evaluated. Because macrophages are antigen-presenting cells that play a key role in the gastrointestinal tract, their response was assessed using the murine macrophage cell line RAW 264.7. Before stimulation, these cells displayed a typical spherical morphology, which was preserved after PBS administration (Figure 4A). In contrast, stimulation with the different treatments induced notable morphological changes, characterized by an elongated, spindle-like shape and the formation of pseudopodia (Figure 4B–D). In this regard, no morphological differences were observed between RAW 264.7 cells stimulated with commercial LPS and those treated with WCs or MVs. Based on these findings, quantitative PCR (qPCR) analysis was performed to evaluate the expression of selected cytokines.

### 2.4. RAW 264.7 Cells Stimulated with WCs or MVs of L. acidophilus Showed Differences in Cytokine Expression

Following the morphological changes observed in RAW264.7 cells after MV administration, the expression levels of the cytokines IL-1β, TNF-α, IL-10, IL-12, and TLR2 were evaluated by quantitative PCR (qPCR).

**Figure 5:**
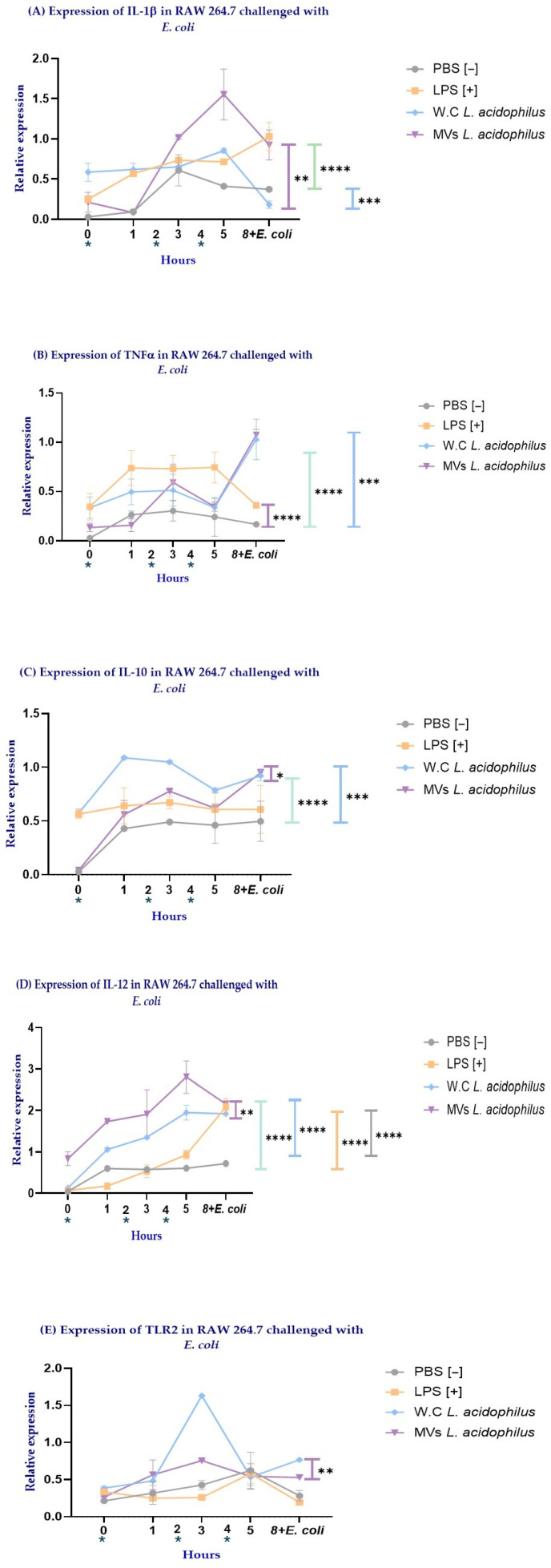
Quantitative PCR (qPCR) analysis of cytokine expression kinetics in RAW 264.7 macrophages. Cells were stimulated with PBS, LPS, whole cells (WCs) of *Lactobacillus acidophilus*, or membrane vesicles (MVs) at 0, 2, and 4 h (*). A baseline sample was collected at 0 h, and additional samples were obtained at 3, 5, and 8 h. At 5 h, macrophages were challenged with *Escherichia coli*. The expression of (**A**) IL-1β, (**B**) TNF-α, (**C**) IL-10, (**D**) IL-12, and (**E**) TLR2 is shown. For statistical purposes, expression values from all time points were considered jointly to assess the overall treatment effect over time. Statistical analysis was performed using one-way ANOVA. Significance is indicated as * *p* ≤ 0.1, ** *p* ≤ 0.05, *** *p* ≤ 0.001, and **** *p* ≤ 0.0001.

**IL-1β** expression peaked at 5 h in cells stimulated with either MVs or WCs. Notably, MV-stimulated cells exhibited significantly higher IL-1β expression compared with WC-stimulated cells (** *p* = 0.0085; purple line) and PBS-treated cells (**** *p* < 0.0001; green line). In contrast, stimulation with WCs resulted in a statistically significant increase in IL-1β expression only when compared with PBS (*** *p* = 0.001) (Figure 5A).

**TNF-α** expression peaked at 8 h in cells stimulated with MVs or WCs, corresponding to 3 h post-*E. coli* challenge. In contrast, LPS-stimulated cells maintained elevated TNF-α expression from 1 to 5 h, followed by a reduction at 8 h after the *E. coli* challenge. Notably, cells stimulated with LPS (purple line) or WCs (green line) exhibited significantly higher TNF-α expression compared with PBS-treated cells (**** *p* < 0.0001 for both). Conversely, MV-stimulated cells showed substantially lower TNF-α expression relative to PBS-treated cells (*** *p* = 0.0006; blue line) (Figure 5B).

**IL-10** expression peaked at 8 h in MV-stimulated RAW 264.7 cells at the end of the *E. coli* challenge. In contrast, cells stimulated with WCs maintained a relatively constant level of IL-10 expression up to 8 h. Notably, WC-stimulated cells exhibited significantly higher IL-10 expression compared with MV-stimulated cells (* *p* = 0.0522; purple line) and also with PBS-treated cells (**** *p* < 0.0001; green line). In contrast, MV-stimulated RAW 264.7 cells showed significantly increased IL-10 expression only when compared with PBS-treated cells (*** *p* = 0.0007, blue line) (Figure 5C).

**IL-12** expression showed a similar pattern in RAW 264.7 cells stimulated with MVs or WCs, reaching peak levels at 8 h at the end of the *E. coli* challenge. Notably, MV-stimulated cells exhibited significantly higher IL-12 expression compared with WC-stimulated cells (** *p* = 0.0043; purple line), PBS-treated cells (**** *p* < 0.0001; green line), and LPS-stimulated cells (**** *p* < 0.0001; blue line). Although WC stimulation did not surpass the effect induced by MVs, WC-stimulated cells still displayed significantly higher IL-12 expression compared with PBS-treated cells (**** *p* < 0.0001; orange line) and LPS-stimulated cells (**** *p* < 0.0001; gray line) (Figure 5D).

**TLR2** expression peaked at 3 h in RAW 264.7 cells stimulated with either MVs or WCs. Notably, only WC-stimulated cells exhibited a statistically significant increase in TLR2 expression compared with PBS-treated cells (** *p* = 0.003; purple line) (Figure 5E).

Overall, the results shown in Figure 5 demonstrate that stimulation of RAW 264.7 macrophages with MVs and WCs from *Lactobacillus acidophilus* induces distinct cytokine expression profiles. MV stimulation was characterized by an early induction of IL-1β, a marked increase in IL-12 expression, a restrained TNF-α response, and moderate but sustained IL-10 expression. This cytokine pattern suggests a selectively regulated immune response that combines proinflammatory and antimicrobial signaling with preserved regulatory control. In contrast, WC stimulation promoted a cytokine profile dominated by IL-10 expression, accompanied by increased TNF-α and limited IL-12 induction, as well as a significant upregulation of TLR2. Collectively, these findings indicate that MVs elicit a more specific and finely tuned immunomodulatory response, whereas WCs induce a broader, predominantly regulatory response, likely mediated through TLR2-dependent pathways.

Accordingly, the next step was to assess the expression of pro- and anti-inflammatory cytokines in RAW 264.7 macrophages under conditions with and without *E. coli* challenge. This approach allowed us to determine whether the immunological profiles induced by MVs and WCs were maintained, enhanced, or differentially regulated in the presence of a pathogenic stimulus, thereby providing insight into their capacity to modulate macrophage responses during bacterial challenge.

### 2.5. E. coli Challenge Enhances the Immunological Profile of Macrophages Stimulated with MVs

The results revealed marked differences in cytokine expression in RAW 264.7 macrophages following *E. coli* challenge, depending on whether cells had been previously stimulated with MVs or WCs.

**Figure 6:**
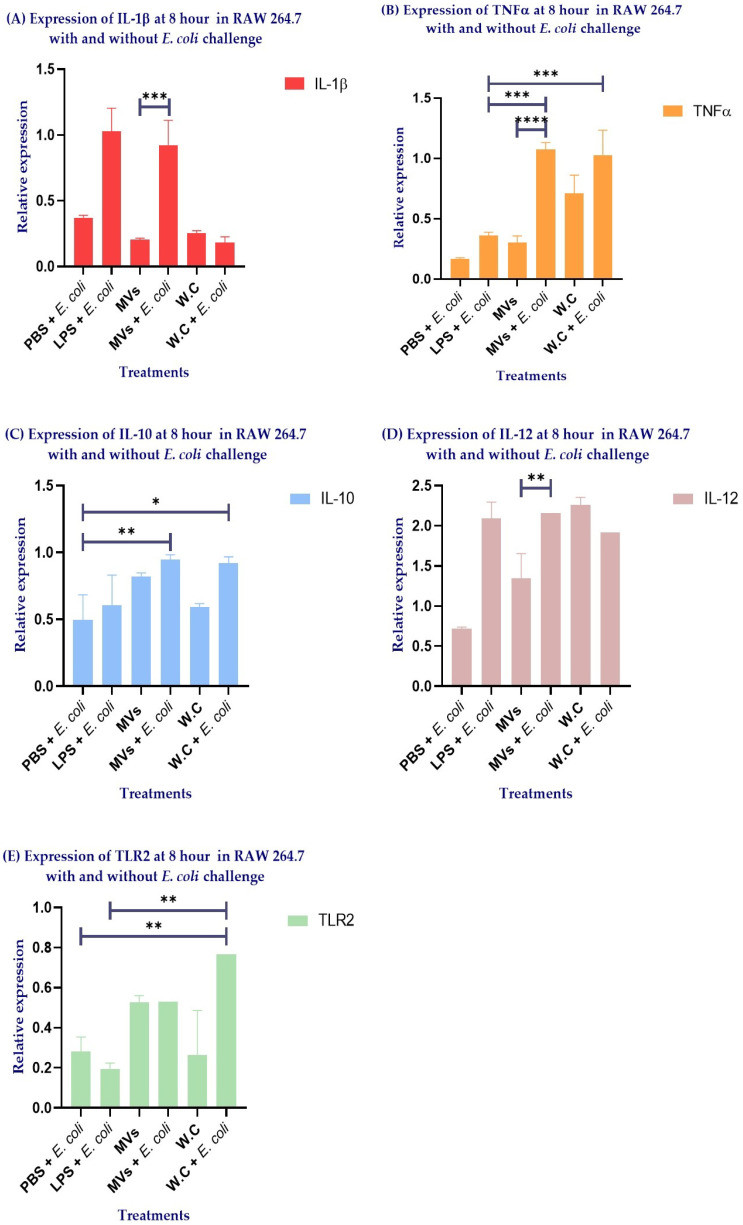
Quantitative PCR (qPCR) analysis of cytokine expression in RAW 264.7 macrophages at 8 h following stimulation with PBS, LPS, whole cells of *Lactobacillus acidophilus* (WCs), or membrane vesicles of *Lactobacillus acidophilus* (MVs), in the presence or absence of *Escherichia coli* challenge. The expression of (**A**) IL-1β, (**B**) TNF-α, (**C**) IL-10, (**D**) IL-12, and (**E**) TLR2 is shown. Statistical analysis was performed using two-way ANOVA. Significance is indicated as * *p* ≤ 0.1, ** *p* ≤ 0.05, *** *p* ≤ 0.001, and **** *p* ≤ 0.0001.

When comparing the MVs + *E. coli* and MVs groups, the bacterial challenge significantly enhanced the expression of the proinflammatory cytokines IL-1β (Figure 6A) and TNF-α (Figure 6B) (*** *p* = 0.0002 and **** *p* < 0.0001, respectively). Notably, this response was accompanied by a selectively regulated cytokine profile, with IL-10 expression maintained (Figure 6C) and IL-12 expression markedly increased (Figure 6D) (** *p* = 0.0013). In addition, the MVs + *E. coli* group exhibited higher TNF-α expression compared with the LPS + *E. coli* group (Figure 6B) (*** *p* = 0.0002) and higher IL-10 expression compared with the PBS + *E. coli* group (Figure 6C) (** *p* = 0.0334).

A distinct pattern was observed in macrophages stimulated with WCs. Following the *E. coli* challenge, WC-stimulated cells showed a tendency toward increased TNF-α, IL-10, and TLR2 expression (Figure 6B,C,E, respectively). However, these changes did not reach statistical significance when compared with the non-challenged WCs group. The concomitant increase in TLR2 (Figure 6E) and IL-10 expression (Figure 6C) suggests that TLR2-mediated signaling may represent a major immunomodulatory pathway activated by WCs. However, the lack of a concomitant rise in IL-12 (Figure 6D) indicates a more generalized and less specialized immunomodulatory response. Consistently, the WC + *E. coli* group exhibited higher TNF-α (Figure 6B) and TLR2 expression (Figure 6E) compared with the LPS + *E. coli* group (*** *p* = 0.0006 and ** *p* = 0.0037, respectively), as well as higher IL-10 (Figure 6C) and TLR2 expression (Figure 6E) compared with the PBS + *E. coli* group (* *p* = 0.0522 and ** *p* = 0.0185, respectively).

Notably, the sustained IL-10 expression observed in the MVs + *E. coli* group (Figure 6C), together with the preserved or enhanced expression of IL-1β, TNF-α, and IL-12 compared with the WC and WC + *E. coli* groups (Figure 6A,B,D, respectively), supports the notion that MVs elicit a more selective and finely tuned immunomodulatory response. This effect appears to be largely independent of TLR2 signaling, further highlighting the distinct and potentially advantageous immunological properties of MVs compared with WCs.

## 3. Discussion

The administration of lactic acid bacteria (LAB) as probiotics has been widely associated with beneficial effects on host health, primarily due to their capacity to enhance immune responses and exert antimicrobial activity [16]. However, because LAB retain the ability to replicate, increasing evidence has highlighted potential risks associated with their use in both immunocompromised individuals [17] and clinically healthy subjects [18]. Consequently, membrane vesicles (MVs) derived from LAB have recently emerged as a promising and safer alternative to conventional antibiotic therapy. Similar to their parental bacteria, these MVs carry bactericidal components that can stimulate the immune response; however, these components are often present at higher concentrations within MVs and, unlike LAB, lack replicative capacity [6].

Therefore, studying these biological agents is a vital strategy to reduce antibiotic resistance while minimizing potential risks to host health.

In this work, transmission electron microscopy (TEM) analysis confirmed that MVs derived from *Lactobacillus acidophilus* exhibit a spherical, double-membrane structure with diameters ranging from approximately 100 to 200 nm, consistent with previous reports for this bacterial genus [19].

Furthermore, both whole cells (WCs) and MVs displayed antimicrobial activity against *Escherichia coli* in a dose-dependent manner, in agreement with previous studies reported for the genus *L. plantarum* [20,21,22]. Notably, the inhibition halos generated by MVs were consistently larger than those produced by WCs at both evaluated concentrations (75 and 100 μg), with the most substantial effect observed at 100 μg of MVs, yielding an inhibition zone of 140 mm (Figure 2). This enhanced activity may be attributed, at least in part, to the smaller size of MVs compared with WCs, as observed by TEM (Figure 1). Their reduced size may facilitate more efficient diffusion through the semi-solid medium, allowing MVs to establish direct contact with the target bacteria, promote membrane fusion, and subsequently release their bioactive components [23,24]. Moreover, the microdilution assay further supported the inhibitory effect observed in the semi-solid medium, demonstrating that MVs retained antibacterial activity across different *E. coli* loads. However, the absorbance values and the calculated percentage of inhibition observed in the WC treatments were likely influenced by the simultaneous growth of *L. acidophilus* WCs and *E. coli* in the microdilution assay. Optical density measurements reflect the total turbidity of the culture and do not discriminate between bacterial species [25]; therefore, the proliferation of viable *L. acidophilus* WCs likely contributed to the overall absorbance, partially masking the inhibitory effect against *E. coli*. This interpretation is supported by the viability assays, which confirmed the growth of *L. acidophilus* WCs on MRS agar, whereas MVs, being non-replicative structures, did not produce colonies (Figure 3). Consequently, only the WC treatments could contribute to increased turbidity in the microdilution assay.

In contrast, MVs represent acellular structures that do not proliferate during the assay, allowing a clearer assessment of their antimicrobial activity. Furthermore, MVs have been reported to contain higher concentrations of bioactive molecules and functional components compared with their parental bacteria [24,26] which may further contribute to their inhibitory capacity. Accordingly, MVs may transport unique factors that enhance antimicrobial activity. Moreover, the composition of MV content is influenced by the surrounding microenvironment and by stimuli that trigger bacterial stress responses [27,28]. In this context, the MVs analyzed in the present study were derived from a field strain of *Lactobacillus acidophilus* isolated from the ileum of free-living rats, an ecological niche characterized by continuous exposure to a diverse array of microorganisms. This environmental pressure may promote the incorporation of a broader range of antigens and antimicrobials into the vesicles, potentially enhancing their microbicidal capacity. In contrast, Dean et al. (2019) characterized MVs from the reference strain *Lactobacillus acidophilus* ATCC 53544 using proteomic analyses and reported the presence of ABC transporters associated with bacteriocin secretion [19]. These antimicrobial peptides exert their effects by inhibiting cell wall synthesis through binding to lipid II and interfering with peptidoglycan transport to the bacterial cell wall. Moreover, due to their net positive charge, bacteriocins can interact with the negatively charged lipopolysaccharide (LPS), leading to a charge imbalance, increased membrane permeability through pore formation, allowing an influx of water, and leakage of intracellular substrates, resulting in bacterial death and growth inhibition [29].

However, Chiba et al. (2024), through proteomic analysis, reported differences in the components present in cell-free supernatant (CFS) compared with MVs isolated from the same supernatants of *Ligilactobacillus salivarius* UO.C249 [30]. In this study, the bacteriocin Abp118 was detected exclusively in the CSF. Nevertheless, the authors demonstrated that MVs exhibited bactericidal activity against *Campylobacter jejuni* ATCC BAA-1153, despite lacking Abp118. This effect was attributed to a higher abundance of components involved in proteolysis, hydrolysis, and peptidase activity, as well as a significantly increased presence of peptidoglycan recognition proteins (PGRPs) and ABC transporters. PGRPs have been shown to induce bacterial death by triggering depolarization and the generation of hydroxyl radicals (OH), thereby inhibiting macromolecular biosynthesis. In Gram-positive bacteria, PGRPs bind to peptidoglycan in the cell wall, leading to wall rupture during cell division. In contrast, in Gram-negative bacteria, they interact directly with the outer membrane, compromising LPS integrity [30].

Similarly, Zhang et al. (2025) describe the transmembrane protein FS25, which exhibits broad antimicrobial activity against both Gram-positive (*Staphylococcus aureus*, *Listeria monocytogenes*) and Gram-negative (*Escherichia coli*, *Salmonella* Enteritidis) bacteria [23]. This effect was shown to be dose-dependent, and the authors proposed that the presence of multiple transmembrane domains may facilitate FS25 anchoring during MV biogenesis, enabling MVs to act as delivery vehicles that exert direct antimicrobial effects following membrane fusion with the target bacterium [23].

Therefore, although WCs have been reported to exert antimicrobial activity through bacteriocins, metabolic products, short-chain fatty acids, and indole compounds [30], MVs possess a distinct, enriched arsenal of bioactive components at higher concentrations, which collectively enhance and amplify their antimicrobial effects [6].

Based on these observations, we hypothesize that one or more of the aforementioned components underlie the superior inhibitory effect of MVs compared with their corresponding whole cells. After demonstrating the antimicrobial effect of *L. acidophilus* MVs, the next step was to stimulate the murine macrophage cell line RAW 264.7 with these MVs to evaluate cytokine expression. In this context, morphological changes were observed following the administration of commercial *Escherichia coli* LPS, as well as MVs and WCs of *L. acidophilus*, with cells transitioning from a regular spherical morphology to a fusiform shape accompanied by the presence of pseudopodia. Consistent with these observations, Xu et al. (2022) reported similar morphological changes in RAW 264.7 cells after stimulation with 10 ng/mL of commercial LPS, which they considered indicative of macrophage activation [31]. Taken together, these findings suggest that the morphological features observed in the present study are likely associated with the activation of this cell line.

In this regard, macrophage activation is essential for mounting an appropriate response to diverse stimuli. Because they can modify their functional phenotype in response to microenvironmental signals, this process is known as polarization. Macrophages can polarize toward an M1 (classically activated) or an M2 (alternatively activated) phenotype, each characterized by distinct markers, cytokines, and chemokines that are directly involved in their functional roles [32].

M1 macrophages are highly effective against intracellular pathogens, and their activation promotes T lymphocyte polarization toward a Th1 profile. Consequently, they secrete proinflammatory cytokines such as TNF-α, IL-6, IL-1β, IL-12, and type I interferons. In contrast, although several M2 subtypes have been described (M2a, M2b, M2c, and M2d), M2 macrophages generally produce cytokines such as IL-4, IL-10, TNF-α, and IL-6. These cells are therefore involved in allergic responses, anti-inflammatory activity, fibrosis induction, Th2 lymphocyte polarization, and immune regulation [32].

Within this functional framework, it is important to consider the intrinsic characteristics of the RAW 264.7 cell line used in this study. As an immortalized murine macrophage model, RAW 264.7 cells exhibit constitutive low-level expression of cytokines and PRRs due to their basal NF-κB activity. This behavior reflects a state of physiological immune readiness rather than true activation [33,34]. Accordingly, PBS-treated cells in our experiments displayed only baseline transcriptional levels, which remained consistently lower than those observed in stimulated groups and did not reach statistical significance.

Against this background, stimulation of RAW 264.7 cells with MVs or WCs resulted in a gradual increase in IL-1β expression, reaching a peak at 5 h. This response may be associated with prior restimulation at 0, 2, and 4 h. In this regard, it has been reported that previous stimulation of pattern recognition receptors (PRRs) of innate immune cells with LAB or non-pathogenic ligands induces a “booster” effect, enabling a faster and more robust response upon a second stimulus, such as an infectious agent [35,36].

In addition, macrophages stimulated with MVs and challenged with *E. coli* demonstrated a selective induction of IL-1β and an absence of a parallel increase in TNF-α, in contrast with WCs, suggesting that MVs promote efficient innate immune activation while limiting excessive inflammatory signaling. IL-1β is a pivotal early proinflammatory cytokine that orchestrates innate immune responses to microbial stimuli, enhances neutrophil recruitment, and promotes pathogen clearance, thereby contributing to host defense (e.g., inflammasome activation leads to antimicrobial responses) [37]. In contrast, TNF-α, although also proinflammatory, is more strongly associated with systemic inflammation and tissue damage when produced in excess, and differential expression of these cytokines has been shown to lead to distinct macrophage activation states and immune outcomes [38]. Therefore, the observed cytokine pattern may favor antimicrobial defense and immune priming without triggering uncontrolled inflammatory cascades that could be detrimental to the host.

In contrast, cells stimulated with WCs before the *E. coli* challenge exhibited a more general immunomodulatory effect, characterized by relatively stable IL-10 expression and a transient TLR2 peak at 3 h. The transient increase in TLR2 expression may activate downstream signaling pathways that modulate cytokine production at the end of the assay.

TLR2 activation has been widely associated with the induction of regulatory and anti-inflammatory responses, including the upregulation of IL-10 in macrophages and dendritic cells (e.g., peptidoglycan-induced IL-10 via TLR2 in APCs) [39]. In line with previous reports, enhanced TLR2 engagement by bacterial cell wall components such as lipoteichoic acid and teichoic acids may account for the pronounced IL-10 expression observed in WC-stimulated cells, and TLR2 ligands have been shown to suppress proinflammatory signaling in immune cells [40,41]. This TLR2–IL-10 axis has been proposed as a key mechanism by which commensal and probiotic bacteria promote immune tolerance and limit excessive inflammation [42].

In contrast, cells stimulated with LPS before the *E. coli* challenge displayed sustained TNF-α expression, indicative of a predominantly proinflammatory and poorly regulated response, which has been associated with tissue damage [38].

Following the *E. coli* challenge, cells pre-stimulated with MVs exhibited increased expression of IL-1β, TNF-α, and IL-12. Notably, this response was accompanied by sustained IL-10 expression throughout the challenge period, with both TNF-α and IL-12 reaching peak levels during this phase. These findings further support the precise and finely coordinated immunomodulatory effect induced by MVs, as discussed above. In this context, the peak in TNF-α expression, together with the presence of IL-10 at the end of the challenge, suggests that TNF-α may play a role more closely associated with tissue repair rather than excessive proinflammatory activity [43,44].

Similarly, following the *E. coli* challenge, MV stimulation induced a marked upregulation of IL-12, indicative of a proinflammatory and Th1-polarizing response, which was accompanied by a more moderate increase in IL-10 expression. This cytokine profile may favor an efficient antimicrobial response while preventing excessive inflammatory damage [45]. The simultaneous induction of IL-12 and IL-10 highlights a balanced immune response, in which proinflammatory signaling is counter-regulated by anti-inflammatory mechanisms to maintain immune homeostasis. This represents an advantage for MVs, as the effectiveness of the immune response against different diseases and pathogens relies on a finely tuned balance between M1 and M2 macrophage polarization, which is essential for an appropriate inflammatory response and subsequent tissue repair [32]. At the same time, this coordinated cytokine response has been described as a hallmark of the immunomodulatory effects exerted by probiotics [46]. It may contribute to the protective activity of *Lactobacillus acidophilus* against enteric pathogens.

In contrast, WC stimulation following the *E. coli* challenge promoted a stronger IL-10 response, accompanied by a moderate increase in IL-12, consistent with a more regulatory or inflammation-resolving macrophage phenotype. In this context, the pronounced induction of IL-10 in WC-stimulated cells may be partially explained by enhanced TLR2 engagement, as TLR2 activation has been associated with anti-inflammatory and regulatory macrophage responses [2,47]. Conversely, the stronger IL-12 response elicited by MVs, despite the lack of significant TLR2 upregulation, suggests that MV-mediated signaling may engage additional pattern recognition receptors or intracellular sensing pathways beyond TLR2. This interpretation is consistent with observations reported by Morishita et al. (2022) for MVs derived from other LAB, who proposed NOD2 and endosomal Toll-like receptors, including TLR3, TLR7, and TLR9, as potential candidates [48].

Together, these findings support a model in which WCs primarily activate TLR2-dependent pathways leading to a regulatory cytokine profile dominated by IL-10. In contrast, MVs induce a more pronounced IL-12-driven response through alternative or complementary signaling routes. Receptors other than TLR2 may underlie the different immunomodulatory effects observed between WCs and their MVs.

The limited interaction between MVs and TLR2 may be related to their compositional characteristics, as MVs are formed by budding of the cytoplasmic membrane and subsequent transit across the peptidoglycan layer, a process that may limit the incorporation of particular cell wall-associated components [6,24,26]. Several studies have reported the absence of peptidoglycan, teichoic acids, and lipoteichoic acids in MVs [49,50], whereas others have described the presence of peptidoglycan-associated components [51,52]. These discrepancies highlight the influence of the biogenesis pathway and the nature of the stimulus on MV cargo composition. Therefore, it cannot be ruled out that MVs derived from *Lactobacillus acidophilus* isolated from the ileum of free-living rats may carry trace amounts of these cell wall components, which may be responsible for their immunomodulatory and antimicrobial effects.

Overall, these findings highlight MVs as a particularly advantageous immunomodulatory strategy compared with WCs. By inducing a finely tuned cytokine profile characterized by the coordinated expression of proinflammatory mediators, such as IL-1β and IL-12, together with regulatory signals like IL-10, MVs promote an effective antimicrobial and Th1-oriented immune response while preventing excessive or uncontrolled inflammation. This balanced activation contrasts with the broader and more TLR2-dependent regulatory profile elicited by WCs and the predominantly proinflammatory response induced by LPS. Importantly, the ability of MVs to engage multiple innate immune sensing pathways without requiring bacterial replication positions them as a safer and more controllable alternative to live probiotics. Collectively, these properties underscore the potential of *L. acidophilus*-derived MVs as next-generation acellular probiotics that can enhance host defense while preserving immune homeostasis.

## 4. Materials and Methods

### 4.1. Bacterial Strains

For this study, we worked with a strain of *Lactobacillus acidophilus* isolated from previous research from the ileum of clinically healthy rats captured in urban settlements of Mexico City. These rodents are resistant to a high concentration of pathogenic microorganisms present in their habitat [53]. Part of this resistance could be associated with their intestinal microbiota, from which we have obtained MVs as the object of study.

In a previous work, membrane vesicles (MVs) from *L. acidophilus* were shown to exert immunostimulatory effects on ovine abomasal explants by reducing the percentage of larval association (L3 *Haemonchus contortus*) and promoting the migration of inflammatory cells to the site of infection. In addition, stimulation of RAW 264.7 macrophages with *L. acidophilus* MVs was previously shown to induce the expression of IL-1β and TNF-α in the first 3 h of the culture [13]. Collectively, these findings supported the selection of *L. acidophilus* as the bacterial model for the current study.

The field strain of *Escherichia coli* was donated by the Institute of Agricultural, Forestry, and Livestock Research (CENID-INIFAP, Mexico) for antimicrobial assays with *L. acidophilus*.

### 4.2. Isolation and Quantification of L. acidophilus MVs

For the isolation of *Lactobacillus acidophilus* MVs, an ultrafiltration- and ultracentrifugation-based protocol was used. Briefly, the *L. acidophilus* strain was cultured on Man–Rogosa–Sharpe (MRS) agar for 24 h at 37 °C under 5% CO_2_. The entire bacterial growth was then transferred to 250 mL of MRS broth and incubated at 37 °C under aerobic conditions for 3.5 h, corresponding to the logarithmic growth phase, in order to enhance MVs production [13,28]. Following incubation, the culture was centrifuged at 9000× *g* for 15 min. The resulting pellet, corresponding to whole cells (WCs), was collected and stored at −4 °C for subsequent assays. The supernatant was sequentially filtered through nitrocellulose membranes with pore sizes of 0.45 μm and 0.22 μm to remove residual cells and debris and subsequently subjected to ultracentrifugation at 150,000× *g* for 3 h at 4 °C. The resulting pellet, corresponding to membrane vesicles (MVs), was resuspended in 500 μL of sterile phosphate-buffered saline (PBS) and stored at −80 °C until further use [12].

Protein concentration was determined using the Bradford assay with a bovine serum albumin (BSA) standard curve and linear regression analysis. All experiments were performed in triplicate using independent biological samples [51].

### 4.3. Transmission Electron Microscopy (TEM) of MVs

To confirm isolation of MVs, as well as to verify vesicle formation, transmission electron microscopy (TEM) was performed. WCs were obtained from *L. acidophilus* cultures grown in MRS medium at 37 °C for 3.5 h (logarithmic phase) under aerobic conditions. MVs were subsequently isolated from the cell-free supernatant of this same culture through ultrafiltration and ultracentrifugation. Samples were deposited onto 200-mesh copper grids coated with formvar (Electron Microscopy Sciences, Hatfield, PA, USA) and carbon-shadowed. A 10 μL sample was applied to the grid and stained with 1% phosphotungstic acid (pH 6.0) (Sigma-Aldrich, St. Louis, MO, USA) for 1 min. Samples were visualized using a JEM 1400 (JEOL, Peabody, MA, USA) transmission electron microscope at the Research and Advanced Studies Center of the National Polytechnic Institute (CINVESTAV), Zacatenco Unit [54].

### 4.4. Antimicrobial Inhibition Assays of Lactobacillus acidophilus MVs and WCs Against Escherichia coli

After confirming the formation and purification of MVs, an antimicrobial inhibition assay was performed. The methodology of Vanegas et al. (2017) was followed with some modifications [55]. Petri dishes were prepared with a base layer of Mueller-Hinton agar (Dibico^®^, Cuautitlán Izcalli, Mexico) and allowed to solidify at room temperature. Subsequently, a layer of previously sterilized and tempered Sulfide, Indole, Motility (SIM) semi-solid medium (BD, Franklyn Lakes, NJ, USA) containing a dilution of *Escherichia coli* (1 × 10^2^) was added. The plates were refrigerated at 4 °C for 2 h [55].

Once the agar had solidified, sensi-disks were loaded with 75 or 100 μg of protein from MVs or WCs to compare their bactericidal effects. WCs were used as the positive control for inhibition, as their antimicrobial activity against *Escherichia coli* has been consistently reported in the literature [56,57,58], whereas sterile PBS served as the negative control. After applying the treatments at different concentrations, the culture plates were incubated for 18–24 h at 37 °C. The inhibition zone diameters were measured in millimeters using a vernier caliper, taking the widest distance across each halo. These assays were performed in triplicate with independent samples.

To complement the inhibition assays, antibacterial activity was also evaluated using a microdilution method. This approach was selected because MVs are complex biological structures containing multiple bioactive molecules whose individual concentrations cannot be precisely determined. Therefore, the assay was designed to assess the inhibitory capacity of MVs against different bacterial loads.

The methodology described by Lee et al. (2021) and Soltani et al. (2022) was followed with minor modifications [20,58]. Briefly, 100 μL of *E. coli* suspensions at different concentrations (10^6^, 10^7^, 10^8^, 10^9^, and 10^10^ CFU/mL) prepared in tryptic soy broth (TSB) were dispensed into 96-well plates. Subsequently, 75 or 100 μg of WCs or MVs were added to the corresponding wells. A positive control containing only *E. coli* suspension and a negative control containing only TSB were included. Plates were incubated at 37 °C for 24 h.

After incubation, optical density was measured at 620 nm (OD_620_), and the percentage of growth inhibition was calculated using the following equation:

Inhibition (%) = [1 − (OD_620_ of growth with MVs or WCs/OD_620_ of *E. coli* growth)] × 100

Finally, 100 μL from each well were plated onto MacConkey agar and MRS agar to confirm bacterial viability. MacConkey agar was used to assess the viability of *E. coli*, whereas MRS agar was used to verify the viability of the *L. acidophilus* WCs. This step was performed to qualitatively confirm the survival of bacteria after treatment [20,58]. All experiments were performed in triplicate.

### 4.5. Stimulation of RAW 264.7 Cells with L. acidophilus WCs or MVs and Challenge with E. coli

To evaluate macrophage stimulation and activation induced by *Lactobacillus acidophilus* WCs or MVs, the methodology of Pérez et al. (2025) was followed with some modifications [13]. The murine macrophage cell line RAW 264.7 (ATCC, Manassas, VA, USA) was seeded in 24-well plates at a density of 1 × 10^5^ cells per well in high-glucose DMEM (4.5 g/L) (Biowest, Nuaillé, France) supplemented with 10% fetal bovine serum (HyClone, Logan, UT, USA). Cells were incubated at 37 °C with 5% CO_2_ for 24 h to allow cell adherence.

Macrophages were stimulated with 10 μg of *L. acidophilus* WCs or MVs. Lipopolysaccharide (LPS) from *Escherichia coli* O111:B4 (2 μg; Sigma-Aldrich, MA, USA) and sterile 1× PBS were included as experimental controls [53]. Stimulations were applied at 0, 2, and 4 h. At 5 h, macrophage morphology was evaluated using an inverted Olympus CKX31 microscope (Olympus Inc., Tokyo, Japan) with a 40× objective (total magnification 400×), and images were captured with an EOS Rebel T7 camera (Canon Inc., Tokyo, Japan) to assess structural changes associated with the different stimuli. For the images acquired with the inverted microscope, spatial calibration was established based on the optical configuration (objective magnification and field number), and scale bars were generated accordingly. After morphological evaluation, cultures were divided into two experimental conditions: a non-challenged group, which received no further treatment, and an *E. coli*-challenged group, to which 10 μL of an *E. coli* suspension (1 × 10^8^ CFU) was added at 5 h. Both groups were then incubated for an additional 3 h, completing a total experimental period of 8 h. Cells were collected at 0, 1, 3, 5, and 8 h for subsequent quantitative PCR (qPCR) analysis. All experiments were performed in triplicate using independent experimental replicates.

### 4.6. qPCR Quantification of IL-1β, TNFα, IL-10, IL-12, and TLR2 in RAW 264.7 Cells Stimulated with L. acidophilus WCs or MVs and Challenged with E. coli

To quantify the expression of TLR2 and selected cytokines, total RNA was extracted from RAW 264.7 cells collected at 0, 1, 3, 5, and 8 h using TRIzol™ Reagent (Thermo Scientific, Waltham, MA, USA), following the manufacturer’s instructions. RNA was purified using the chloroform–isopropanol–ethanol precipitation method as previously described [53]. The resulting RNA pellet was resuspended in 50 μL of RNase-free water, and RNA concentration and purity were determined by spectrophotometry using a NanoDrop Lite system (Thermo Scientific, Waltham, MA, USA).

Complementary DNA (cDNA) was synthesized from the purified RNA using the FastGene Scriptase Basic cDNA Synthesis Kit (Nippon Genetics, Tokyo, Japan), according to the manufacturer’s protocol. The concentration of the synthesized cDNA was quantified by spectrophotometry prior to qPCR analysis.

Gene-specific primers for TLR2, IL-1β, TNF-α, IL-10, and IL-12 were designed based on published murine sequences obtained from GenBank (TLR2, NM_011905.3; IL-1β, NM_008361.4; TNF-α, NM_001278601.1; IL-10, NM_010548.2; IL-12, NM_001303244.1). Primer design was performed using Primer3 software (v0.4.0), and sequence alignment and verification were carried out using BioEdit (v7.2.5; Ibis Bioscience, Carlsbad, CA, USA). All primers were synthesized by T4Oligo (Irapuato, Guanajuato, Mexico), and their sequences are listed in Table 1.

Quantitative PCR (qPCR) reactions were performed in triplicate using independent samples for each target gene. Each reaction contained 10 ng of cDNA and RealQ Plus Master Mix Green without ROX (AMPLIQON, Odense, Denmark). Amplification was carried out using an Agilent Mx3005P real-time PCR system (Agilent Technologies, Santa Clara, CA, USA).

The housekeeping gene hypoxanthine–guanine phosphoribosyltransferase (HPRT) was used as an internal reference control. The amplification protocol consisted of an initial enzyme activation step at 95 °C for 15 min, followed by 40 cycles of denaturation at 95 °C for 30 s, annealing at 50 °C for 30 s, and extension at 72 °C for 30 s. Annealing temperatures for individual cytokines varied and are detailed in Table 1. Amplification and melting (dissociation) curves were generated to confirm primer specificity and the absence of nonspecific amplification (Figure S1).

Relative gene expression levels were calculated using the comparative ΔΔCt method, applying the following equations:

Cp (sample) − Cp (HPRT) = ΔCp

ΔCp (sample) − ΔCp (calibrator) = ΔΔCp

Relative quantity = 2 − ΔΔCp

Subsequently, the logarithmic transformation of the relative values obtained was performed using the base 10 logarithm ([2 − ΔΔCp (Log 10)]) [59,60].

### 4.7. Statistical Analysis

Data were analyzed using a one-tailed Student’s *t*-test and analysis of variance (ANOVA), followed by Tukey’s test. Statistical analyses were conducted using GraphPad Prism 8.0.2 (GraphPad, San Diego, CA, USA). Differences were considered significant when *p* ≤ 0.1 (*), *p* ≤ 0.05 (**), *p* ≤ 0.001 (***), and *p* ≤ 0.0001 (****).

## 5. Conclusions

In conclusion, MVs derived from *Lactobacillus acidophilus* isolated from the ileum of free-living rats exhibited both direct bactericidal activity against *E. coli* and the ability to modulate macrophage immune responses. Compared with WCs, MVs induced a more selective and balanced cytokine profile, characterized by the coordinated expression of proinflammatory and regulatory mediators. This dual antimicrobial and immunomodulatory capacity highlights the potential of *L. acidophilus*-derived MVs as a promising acellular probiotic for the prevention and control of infectious diseases, offering a novel and potentially safer alternative to conventional probiotic-based strategies.

## Figures and Tables

**Table 1:** Primer Sequences and Annealing Parameters for IL-1β, TNFα, IL-10, IL-12 and TLR2.

Citokines	Secuences	Temperature of Alignment	Expected Size
IL-1β	Fw: GGTGTGTGACGTTCCCATTA	62 °C	170 pb
	Rv: CGTTGCTTGGTTCTCCTTGT		
TNFα	Fw: TATGGCTCAGGGTCCAACTC	59 °C	174 pb
	Rv: CTCCCTTTGCAGAACTCAGG		
IL-10	Fw: GCCTTATCGGAAATGATCC	56 °C	176 pb
	Rv: TCCACTGCCTTGCTCTTATT		
IL-12	Fw: ACAGCACCAGCTTCTTCATC	57 °C	165 pb
	Rv: GCTGGATTCGAACAAAGAACT		
TLR2	Fw: CTCCCACTTCAGGCTCTTTG	61 °C	223 pb
	Rv: GAAGTCAGGAACTGGGTGGA		

## Data Availability

The data presented in this study are openly available in Harvard Dataverse: https://doi.org/10.7910/DVN/1A0XWY.

## Supplementary Materials

The following supporting information can be downloaded at: https://www.mdpi.com/article/10.3390/ijms27062764/s1.

## Abbreviations

The following abbreviations are used in this manuscript:

ANOVA	Analysis of variance
APCs	Antigen-presenting cells
BSA	Bovine serum albumin
cDNA	Complementary DNA
CFS	Cell-free supernatant
DMEM	Dulbecco’s modified Eagle medium
HPRT	Hypoxanthine–guanine phosphoribosyltransferase
IL-1β	Interleukin 1 beta
IL-4	Interleukin 4
IL-6	Interleukin 6
IL-10	Interleukin 10
IL-12	Interleukin 12
LAB	Lactic acid bacteria
LPS	Lipopolysaccharide
MRS	Man–Rogosa–Sharpe medium
MVs	Membrane vesicles
NOD2	Nucleotide-binding oligomerization domain-containing protein 2
OH	Hydroxyl radical
PBS	Phosphate-buffered saline
PGRPs	Peptidoglycan recognition proteins
PRRs	Pattern recognition receptors
qPCR	Quantitative polymerase chain reaction
RNA	Ribonucleic acid
SIM	Sulfide, indole, motility medium
TEM	Transmission electron microscopy
TLR2	Toll-like receptor 2
TLR3	Toll-like receptor 3
TLR7	Toll-like receptor 7
TLR9	Toll-like receptor 9
TNF-α	Tumor necrosis factor alpha
WCs	Whole cells
