# Supplementary material for: Membrane Vesicles from Lactobacillus acidophilus Promote Superior Cytokine Modulation and Antimicrobial Signaling Compared with Their Whole Cells in RAW 264.7 Macrophages
Source: Int J Mol Sci. 2026 Mar 18;27(6):2764. doi: 10.3390/ijms27062764 (PMC13026561; doi:10.3390/ijms27062764)
Supplement: Supplementary file 1 [file ijms-27-02764-s001.zip › ijms-4149470-supplementary.pdf]

Supplementary Materials:

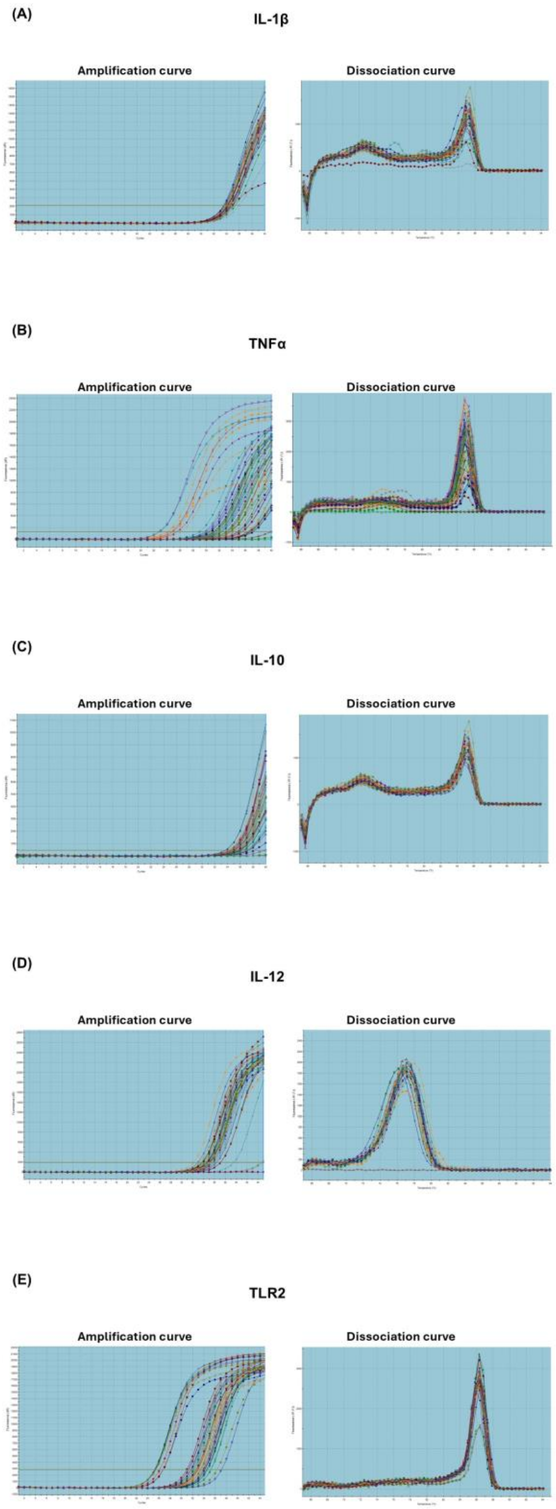

Supplementary Figure S1. Dissociation and amplification curves for validation and specificity of the primers synthesized for qPCR. (A). IL-1β, (B) TNF α, (C) IL-10, (D) IL-12 and (E) TLR2.
